# Supplementary material for: Engineering recurrent neural networks from task-relevant manifolds and dynamics
Source: PLoS Comput Biol. 2020 Aug 12;16(8):e1008128. doi: 10.1371/journal.pcbi.1008128 (PMC7446915; doi:10.1371/journal.pcbi.1008128)
Supplement: S1 Fig — a) Drift values around a planar ring with different numbers of fixed points (unity line: dashed). b) Same as (a) for a planar ring with 6 fixed points and a drift function with difference baseline values. Results indicate that the measured drift values closely follow the target values. (PDF) [file pcbi.1008128.s001.pdf]

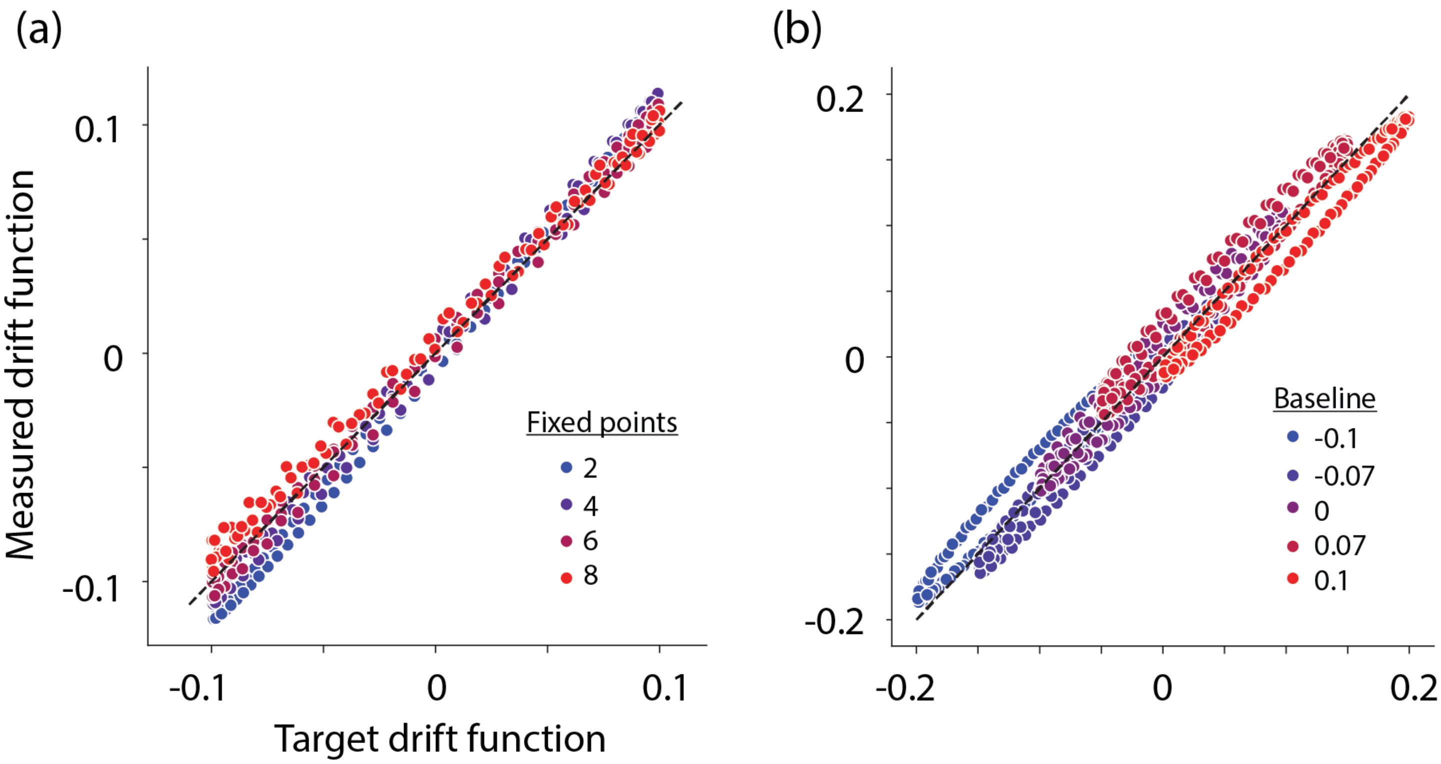

S1 Fig. Comparison of the target and measured drift functions. a) Drift values around a planar ring with different numbers of fixed points (unity line: dashed). b) Same as (a) for a planar ring with 6 fixed points and a drift function with difference baseline values. Results indicate that the measured drift values closely follow the target values.
