# Supplementary material for: Engineering recurrent neural networks from task-relevant manifolds and dynamics
Source: PLoS Comput Biol. 2020 Aug 12;16(8):e1008128. doi: 10.1371/journal.pcbi.1008128 (PMC7446915; doi:10.1371/journal.pcbi.1008128)
Supplement: S2 Fig — To test how the network responds to noise, we added independent Gaussian noise to each unit of a 400-unit RNN at every timestep for 5 seconds. The RNN was constructed for O(2,6). a) The input-output mapping of each unit with 4 levels of noise. The standard deviation of noise (σ) is shown on top. The shaded region shows the expected shift in the output for signals deviating one standard deviation from the mean. b) The initial state (blue), intermediate state (black), and end states (red) of network activity plotted in a subspace spanned by the first three principal components for 20 evenly spaced initializations around the ring. For low-noise regimes, the network converges on the desired fixed points and stays confined to the ring. Intermediate noise causes the trajectories to diffuse slightly but remain close to the fixed points. For the highest noise, trajectories are no longer confined to the fixed points. Attempts at mapping the level of network noise to noise in a corresponding drift-diffusion model were not successful. c) A circular histogram of decoded end states around the ring after 5 seconds across 100 evenly spaced initializations. The six fixed points are located every 60 degrees. (PDF) [file pcbi.1008128.s002.pdf]

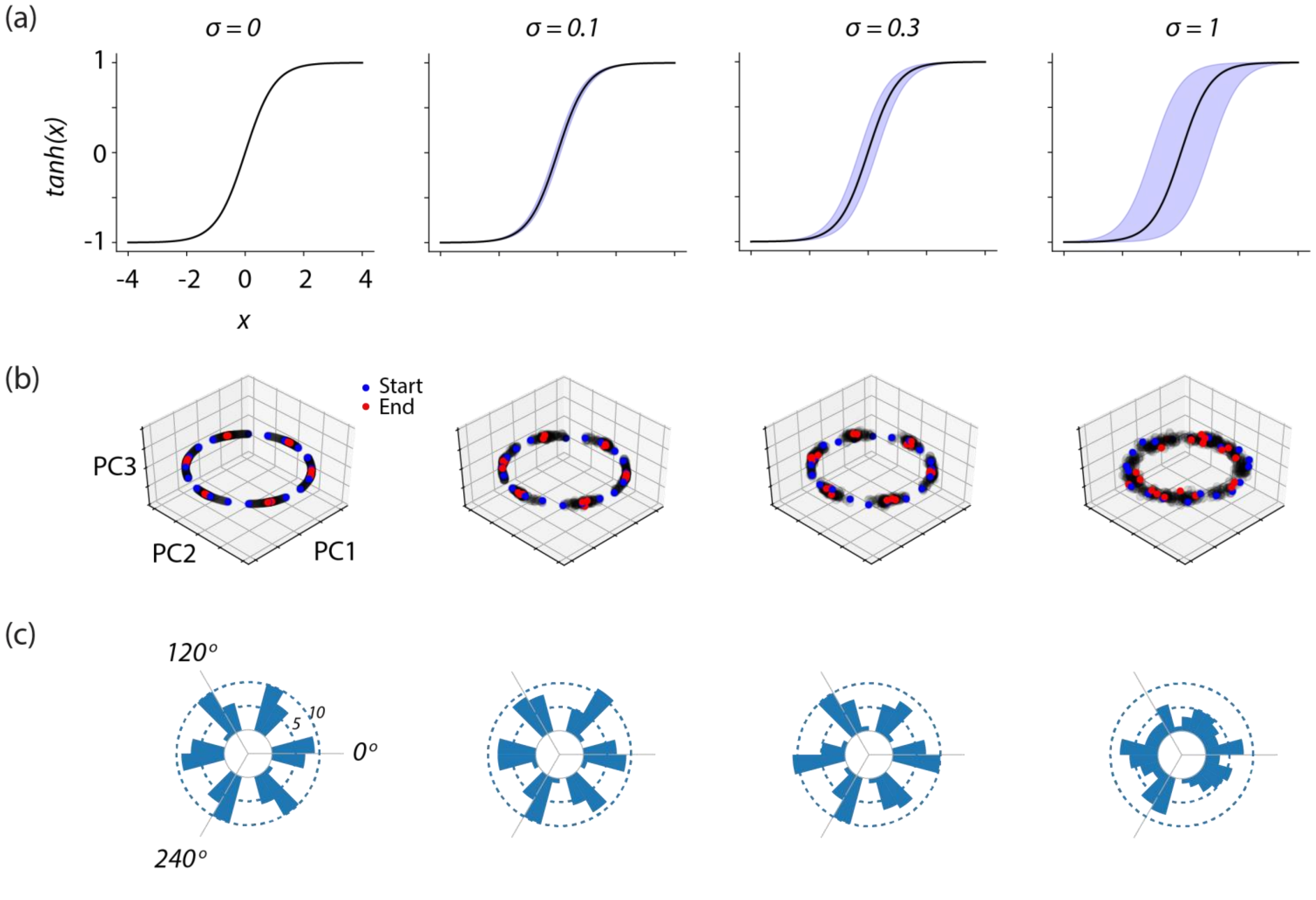

S2 Fig. Performance with respect to internal noise. To test how the network responds to noise, we added independent Gaussian noise to each unit of a 400-unit RNN at every timestep for 5 seconds. The RNN was constructed for O(2,6). a) The input-output mapping of each unit with 4 levels of noise. The standard deviation of noise (σ) is shown on top. The shaded region shows the expected shift in the output for signals deviating one standard deviation from the mean. b) The initial state (blue), intermediate state (black), and end states (red) of network activity plotted in a subspace spanned by the first three principal components for 20 evenly spaced initializations around the ring. For low-noise regimes, the network converges on the desired fixed points and stays confined to the ring. Intermediate noise causes the trajectories to diffuse slightly but remain close to the fixed points. For the highest noise, trajectories are no longer confined to the fixed points. Attempts at mapping the level of network noise to noise in a corresponding drift-diffusion model were not successful. c) A circular histogram of decoded end states around the ring after 5 seconds across 100 evenly spaced initializations. The six fixed points are located every 60 degrees.
