# Supplementary material for: Engineering recurrent neural networks from task-relevant manifolds and dynamics
Source: PLoS Comput Biol. 2020 Aug 12;16(8):e1008128. doi: 10.1371/journal.pcbi.1008128 (PMC7446915; doi:10.1371/journal.pcbi.1008128)
Supplement: S3 Fig — a) Example of latent tuning functions for a hypersphere in an 8-dimensional subspace (2 sinusoidal and 6 von Mises functions). b) Due to normalization across latent tuning functions, the ring always lies on the surface of a hypersphere. Left: A high-dimensional ring over a hypersphere with 2 sinusoids and 6 von Mises functions with width parameter, κ = 2, plotted in a coordinate system made of the three first latent tuning functions (c1, c2 and c3). Right: The same ring when the von Mises functions are infinitely broad (κ = 0). c) Example single-unit tuning functions from a network constructed using the latent tuning functions in a). Different colors represent different single units. d) The total ring length as a function of the embedding dimension, for different width parameters of the von Mises function. For broad tuning (low κ), increasing the embedding dimension shortens the ring. Note that this is due to our normalization scheme, which forces the ring to lie on a hypersphere (see right side of panel b). Conversely, increasing the embedding dimension of the ring when the tuning functions are relatively narrow will monotonically lengthen the ring. This is reminiscent of the theoretical result that increasing the density of narrow tuning functions tiling the stimulus space could increase Fisher information [33]. (PDF) [file pcbi.1008128.s003.pdf]

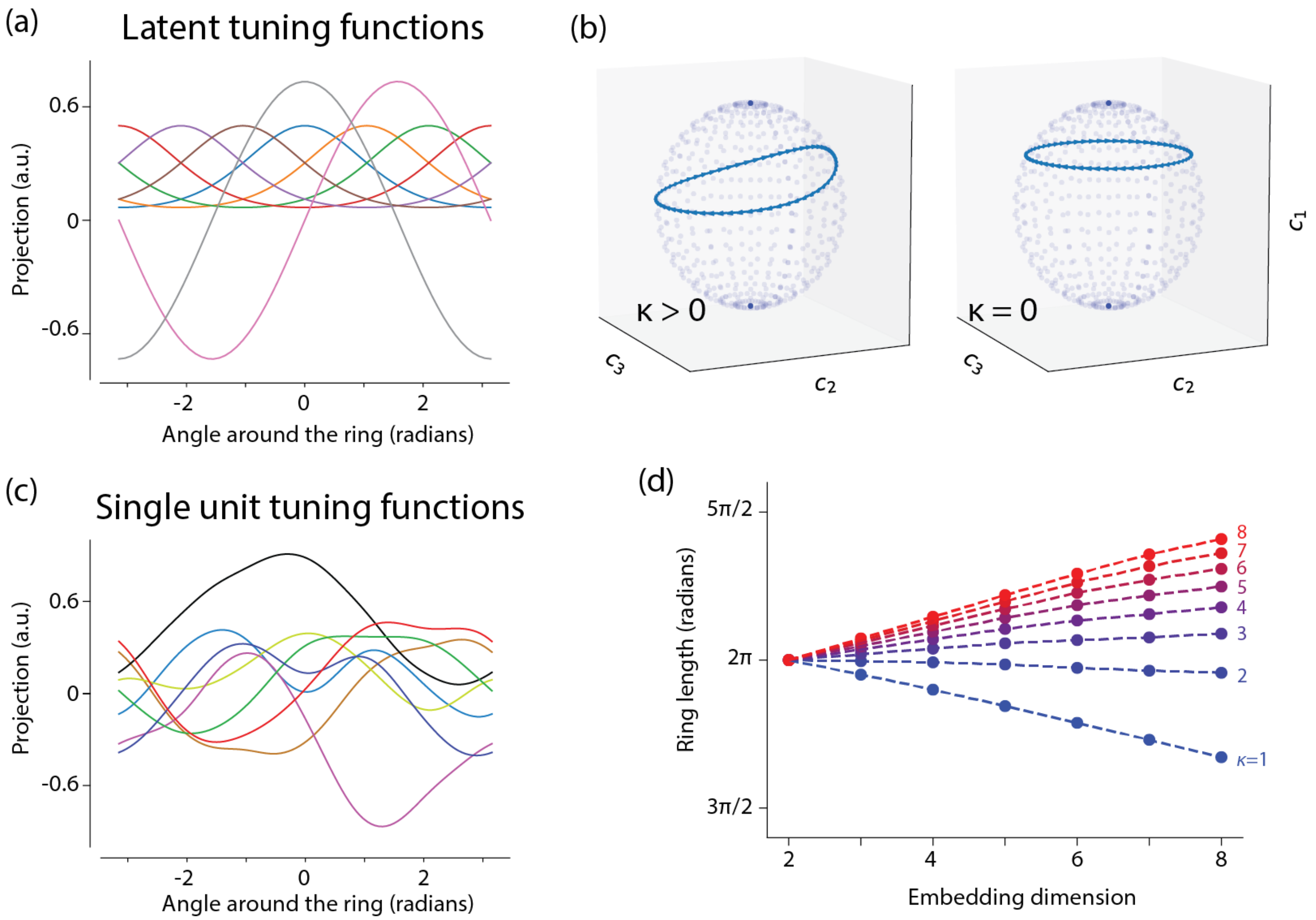

S3 Fig. High-dimensional rings on hyperspheres. a) Example of latent tuning functions for a hypersphere in an 8-dimensional subspace (2 sinusoidal and 6 von Mises functions). b) Due to normalization across latent tuning functions, the ring always lies on the surface of a hypersphere. Left: A high-dimensional ring over a hypersphere with 2 sinusoids and 6 von Mises functions with width parameter, κ=2, plotted in a coordinate system made of the three first latent tuning functions (c_1, c_2 and c_3). Right: The same ring when the von Mises functions are infinitely broad (κ=0). c) Example single-unit tuning functions from a network constructed using the latent tuning functions in a). Different colors represent different single units. d) The total ring length as a function of the embedding dimension, for different width parameters of the von Mises function. For broad tuning (low κ), increasing the embedding dimension shortens the ring. Note that this is due to our normalization scheme, which forces the ring to lie on a hypersphere (see right side of panel b). Conversely, increasing the embedding dimension of the ring when the tuning functions are relatively narrow will monotonically lengthen the ring. This is reminiscent of the theoretical result that increasing the density of narrow tuning functions tiling the stimulus space could increase Fisher information [33].
