# Supplementary material for: Engineering recurrent neural networks from task-relevant manifolds and dynamics
Source: PLoS Comput Biol. 2020 Aug 12;16(8):e1008128. doi: 10.1371/journal.pcbi.1008128 (PMC7446915; doi:10.1371/journal.pcbi.1008128)
Supplement: S4 Fig — We constructed 400-unit RNNs to embed either a 2-dimensional (top) or an 8-dimensional ring (bottom) of radius 12 and infinitely many fixed points (continuous attractor). We then simulated the RNNs from 100 random initializations. The initial conditions were drawn randomly from a spherical multivariate Gaussian distribution with a standard deviation of 12 in all dimensions. a) The initial state (blue) and end states (red) after 2 seconds of network activity plotted in a subspace spanned by the first three principal components for the two RNNs. b) The deviation (Eq 17) of neural states as a function of time during each simulation normalized by the radius. Initial deviations are very large, but the network state rapidly approaches the region of the state space near the ring. For the 2-dimensional ring (top), the end states coincide with the ring, suggesting that the ring acts as a global attractor. For the 8-dimensional ring, end states deviated more from the target manifold, suggesting that higher-dimensional rings are less robust. (PDF) [file pcbi.1008128.s004.pdf]

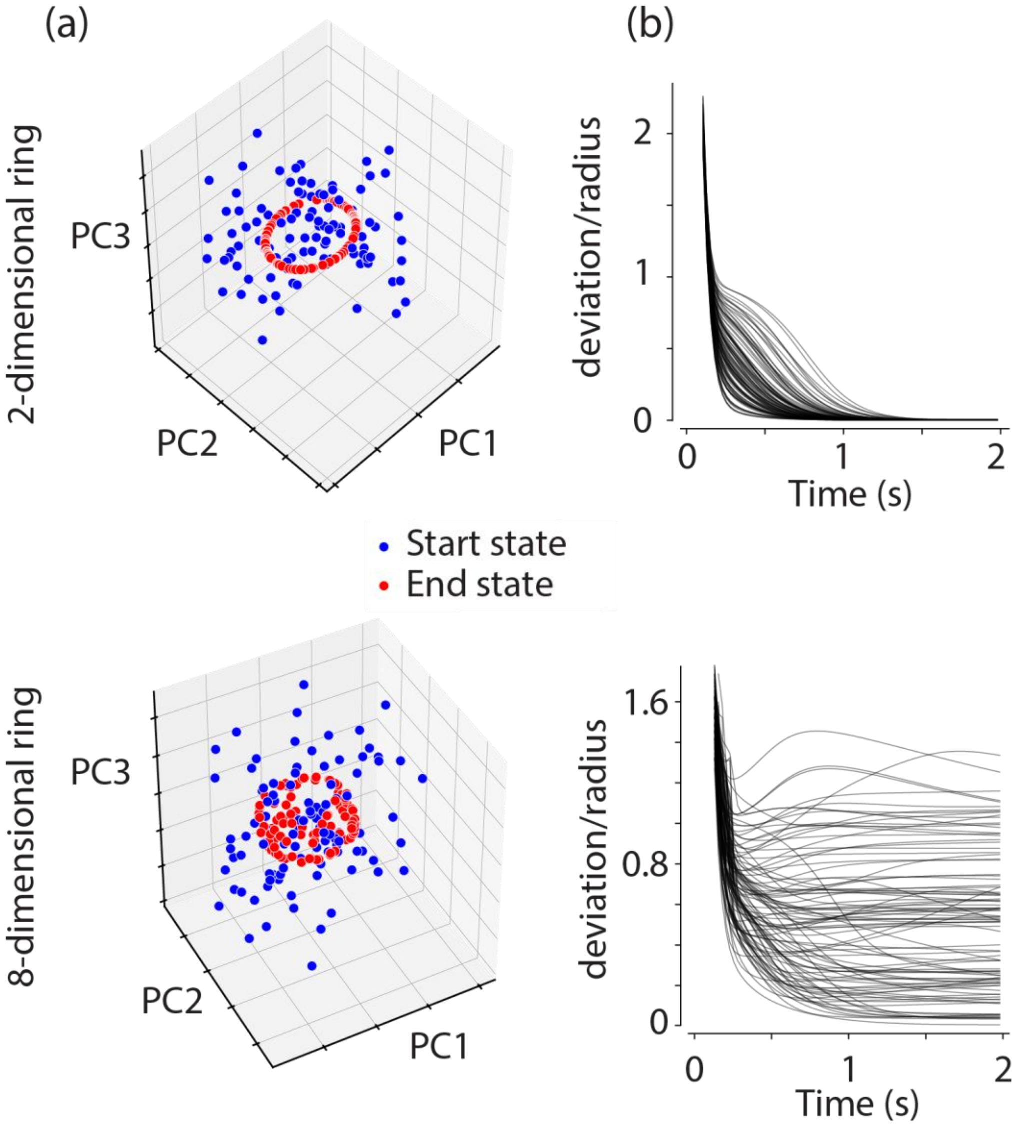

S4 Fig. Low-D RNNs have a more robust basin of attraction. We constructed 400-unit RNNs to embed either a 2-dimensional (top) or an 8-dimensional ring (bottom) of radius 12 and infinitely many fixed points (continuous attractor). We then simulated the RNNs from 100 random initializations. The initial conditions were drawn randomly from a spherical multivariate Gaussian distribution with a standard deviation of 12 in all dimensions. a) The initial state (blue) and end states (red) after 2 seconds of network activity plotted in a subspace spanned by the first three principal components for the two RNNs. b) The deviation (Eq. 17) of neural states as a function of time during each simulation normalized by the radius. Initial deviations are very large, but the network state rapidly approaches the region of the state space near the ring. For the 2-dimensional ring (top), the end states coincide with the ring, suggesting that the ring acts as a global attractor. For the 8-dimensional ring, end states deviated more from the target manifold, suggesting that higher-dimensional rings are less robust.
