# Supplementary material for: Engineering recurrent neural networks from task-relevant manifolds and dynamics
Source: PLoS Comput Biol. 2020 Aug 12;16(8):e1008128. doi: 10.1371/journal.pcbi.1008128 (PMC7446915; doi:10.1371/journal.pcbi.1008128)
Supplement: S5 Fig — We analyzed how the number of setpoints impacts the degree to which engineered RNNs can capture target manifolds and dynamics. We constructed the RNN to have O(8,6) with drift amplitude of 0.1 rad/s over a ring of radius 12. a) Violin plot showing the mean squared error between the measured and target drift function as a function of number of setpoints. Inset: Target drift function (red) and the corresponding measured drift function with 6 setpoints (black). b) Violin plot showing the deviation (Eq 17) of neural states as a function of number of setpoints normalized by the radius. Results are shown for 20 simulations over a 5 second period, with deviation calculated from 20 evenly spaced initializations. Inset: The initial state (blue), intermediate states (gray), and end states (red) of a network constructed from 6 setpoints, plotted in a subspace spanned by the first three principal components. (PDF) [file pcbi.1008128.s005.pdf]

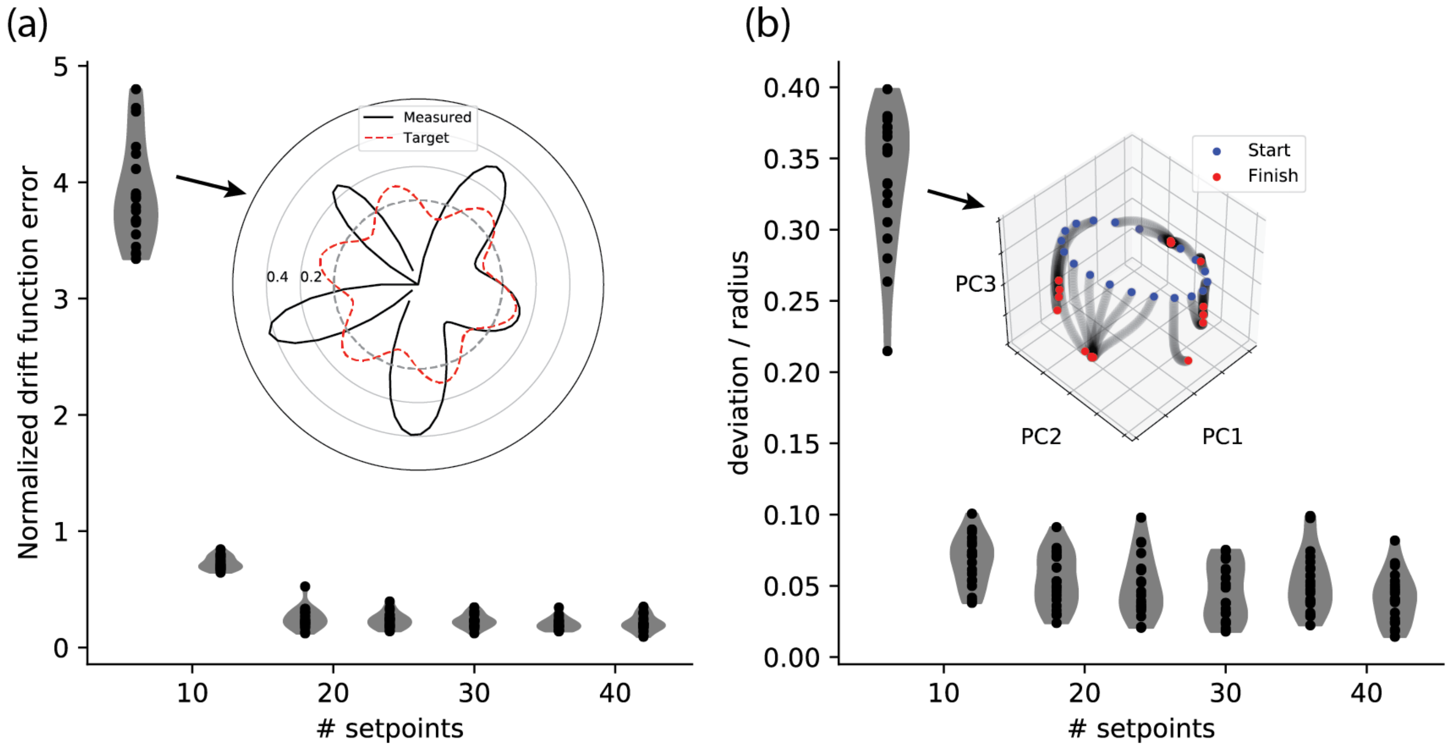

S5 Fig. Performance with respect to the number of setpoints. We analyzed how the number of setpoints impacts the degree to which engineered RNNs can capture target manifolds and dynamics. We constructed the RNN to have O(8,6) with drift amplitude of 0.1 rad/s over a ring of radius 12. a) Violin plot showing the mean squared error between the measured and target drift function as a function of number of setpoints. Inset: Target drift function (red) and the corresponding measured drift function with 6 setpoints (black). b) Violin plot showing the deviation (Eq. 17) of neural states as a function of number of setpoints normalized by the radius. Results are shown for 20 simulations over a 5 second period, with deviation calculated from 20 evenly spaced initializations. Inset: The initial state (blue), intermediate states (gray), and end states (red) of a network constructed from 6 setpoints, plotted in a subspace spanned by the first three principal components.
