# Supplementary material for: Engineering recurrent neural networks from task-relevant manifolds and dynamics
Source: PLoS Comput Biol. 2020 Aug 12;16(8):e1008128. doi: 10.1371/journal.pcbi.1008128 (PMC7446915; doi:10.1371/journal.pcbi.1008128)
Supplement: S6 Fig — The radius of the ring is equal to the magnitude of the projection vectors. We quantified how the radius changes the operating point of units with respect to their saturation point, and impacts the robustness of a 400-unit RNN engineered for O(8,6). a) Projections of the first latent vector in the neural state space (abscissa) passed through a hyperbolic tangent function for rings with different radii (ordinate). In rings with a large radius, more units operate near the saturation regime of their activation function. b) Violin plot showing the mean deviation (Eq 17) as a function of radius. For each radius value, 30 networks were simulated. Mean deviation was computed across 20 trajectories over 2 seconds. The line shows the means of the distributions of deviations for each radius. (PDF) [file pcbi.1008128.s006.pdf]

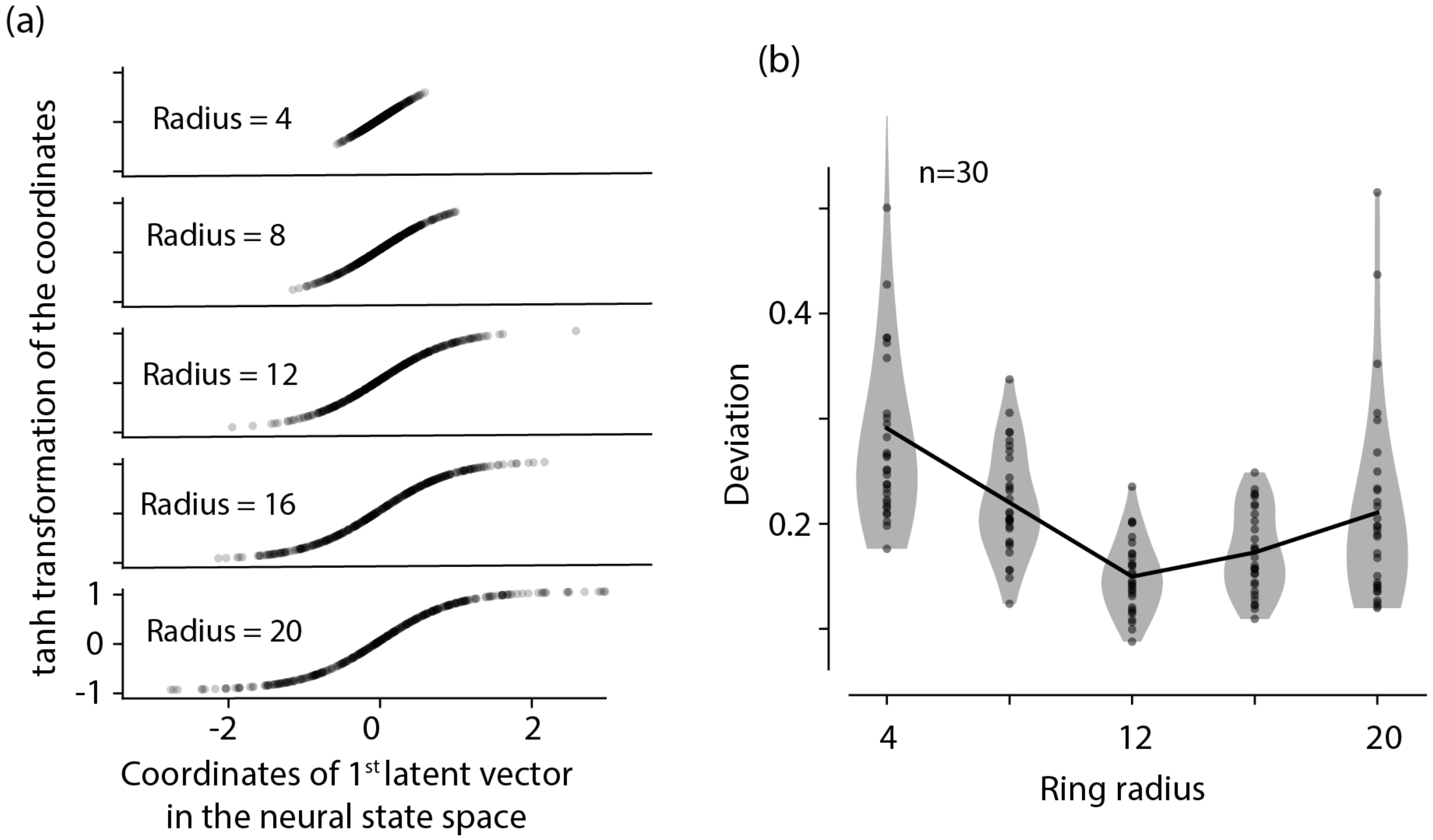


**S6 Fig. Ring radius, unit saturation, and deviation.** The radius of the ring is equal to the magnitude of the projection vectors. We quantified how the radius changes the operating point of units with respect to their saturation point, and impacts the robustness of a 400-unit RNN engineered for O(8,6). a) Projections of the first latent vector in the neural state space (abscissa) passed through a hyperbolic tangent function for rings with different radii (ordinate). In rings with large radii, more units operate near the saturation regime of their activation function. b) Violin plot showing the mean deviation (Eq. 17) as a function of radius. For each radius value, 30 networks were simulated. Mean deviation was computed across 20 trajectories over 2 seconds. The line shows the means of the distributions of deviations for each radius.
