# Supplementary material for: Engineering recurrent neural networks from task-relevant manifolds and dynamics
Source: PLoS Comput Biol. 2020 Aug 12;16(8):e1008128. doi: 10.1371/journal.pcbi.1008128 (PMC7446915; doi:10.1371/journal.pcbi.1008128)
Supplement: Attachment — Submitted filename: EP_MJ_Review_Response.docx [file pcbi.1008128.s008.docx]

We thank the reviewers for their careful reading of our manuscript and for their insightful comments. We have submitted a revised manuscript that addresses their concerns. In the following, we provide a detailed response to each point raised by the reviewers and describe how we changed the manuscript to accommodate the various suggestions. The reviewers’ comments are quoted in blue, followed by our responses.

––––––––––––––––––––––––––––––––––––––––––––––––––––––––––––––––––––––––––––

**Reviewer #1**

Minor revisions:

1. One question that many readers will want to know the answer to is how robust the method is, a point which the authors note twice themselves (line 212 "such large vectors caused unpredictable network behavior" and line 397 "if the dynamics are fast enough that linear approximations no longer work"). Obviously, answering this question will be dependent on the task, number of neurons, etc. so this can't be answered exhaustively, but I think just giving the reader a taste of the robustness is important. For example, answering that question within the confines of the ring attractor model, with whatever standard parameters were used throughout the paper would be sufficient. Two ways I could imagine answering this question would be to measure the relaxation dynamics to increasingly larger levels of internal noise (as was already done), or to measure the relaxation dynamics to initializations way off the ring (although I leave it to the authors to decide the best way to illustrate this point).

The reviewer asks that we give the reader a taste of robustness to noise and initialization for the ring attractor. We think that this is an excellent suggestion and have revised the manuscript accordingly.

First, we used the 2-dimensional ring network to assess the effect of noise. We introduced independent noise to each network unit, controlling the standard deviation. For various levels of noise, we compared the noise standard deviation to the unit activation function, showed state space trajectories, and plotted a histogram of the network’s end position on the ring after 5 seconds of simulation time. We showed how increasing the noise level gradually degrades the network’s performance until the six fixed points are no longer apparent. This result is shown in a new supplementary figure (Figure S2).

Next, we looked at how robust networks are to initial conditions. Our simulations indicated that the robustness was dependent on the embedding dimension. Accordingly, we have added a supplemental figure (Figure S4) that demonstrates robustness to initial conditions for networks with an embedding dimensionality of 2 and 8. For the 2-dimensional case, all trajectories quickly converged to the ring, suggesting that the ring has a large basin of attraction. However, for the 8-dimensional case, many trajectories did not converge to the ring.

For the reviewer’s convenience, copies of these figures with their captions are included below:

**Figure S2. Performance with respect to internal noise.** To test how the network responds to noise, we added independent Gaussian noise to each unit of a 400-unit RNN at every timestep for 5 seconds. The RNN was constructed for O(2,6). a) The input-output mapping of each unit with 4 levels of noise. The standard deviation of noise (*σ*) is shown on top. The shaded region shows the expected shift in the output for signals deviating one standard deviation from the mean. b) The initial state (blue), intermediate state (black), and end states (red) of network activity plotted in a subspace spanned by the first three principal components for 20 evenly spaced initializations around the ring. For low-noise regimes, the network converges on the desired fixed points and stays confined to the ring. Intermediate noise causes the trajectories to diffuse slightly but remain close to the fixed points. For the highest noise, trajectories are no longer confined to the fixed points. c) A circular histogram of decoded end states around the ring after 5 seconds across 100 evenly spaced initializations. The six fixed points are located every 60 degrees.

**Figure S4. Low-D RNNs have a more robust basin of attraction.** We constructed 400-unit RNNs to embed either a 2-dimensional (top) or an 8-dimensional ring (bottom) of radius 12 and infinitely many fixed points (continuous attractor). We then simulated the RNNs from 100 random initializations. The initial conditions were drawn randomly from a spherical multivariate Gaussian distribution with a standard deviation of 12 in all dimensions. a) The initial state (blue) and end states (red) after 2 seconds of network activity plotted in a subspace spanned by the first three principal components for the two RNNs. b) The deviation (equation 17) of neural states as a function of time during each simulation normalized by the radius. Initial deviations are very large, but the network state rapidly approaches the region of the state space near the ring. For the 2-dimensional ring (top), the end states coincide with the ring, suggesting that the ring acts as a global attractor. For the 8-dimensional ring, end states deviated more from the target manifold, suggesting that higher-dimensional rings are less robust.

One additional benchmark that might be useful would be to compare the robustness to noise or initialization off-manifold to methods (such as NEF) that use the global dynamics equations (as the authors noted). This might be a more expansive revision that the authors are willing to undertake. I leave it to the authors to decide if they wish to include this, but I personally feel it would strengthen the paper and better allow readers to understand the relationship of this new method to existing methods.

The reviewer has made an optional recommendation that we analyze the robustness of other methods with respect to noise and initialization. The main challenge in addressing this point is that different methods have different strengths and weaknesses and comparing them on an arbitrary choice of task (e.g. ring attractor) would provide a skewed picture of how to arbitrate between them for a new problem. Of course, one can instead go all the way and analyze robustness for a large database of networks, tasks, and parameters, but we feel that such an undertaking, as valuable as it will surely be, is beyond the scope of our manuscript.

General comments:

Broadly, the paper is well-written and clear. However, also, broadly, I would categorize the writing as being targeted to readers with a high-level of prior knowledge about the subject. Personally, I would urge the authors to "hand-hold" the reader a bit more, to increase the chances that a reader without a super high-level of subject familiarity will read and understand this very important work. (This is merely a suggestion, not a requirement). It's difficult to give an exhaustive lists of these issues, but I will provide two, to hopefully clarify this:

1). The introduction can be a bit jargony (e.g. notice the number of quoted phrases- "latent task space", "latent task manifold", "latent task dynamics"). This is not a problem for a fairly acquainted reader, but the authors might want to unpack some of these ideas a bit more.

Thank you for this suggestion. We think that it is important that the material is broadly accessible. We made a significant revision to address this comment. First, we rewrote the entire Introduction so that it is broadly accessible (nearly all jargon removed). Second, we augmented the Results section with two introductory subsections in which we “hand-hold” the reader as we carefully introduce the reader to various terms such as “manifold”.

2). The last two sections are pretty unmotivated and I was often left wanting more exposition here ("Rings embedded..." and "Limitations of RNN..."). To me, this is unfortunate because here you are really paying off the development of your method- you can use it to construct neural systems that represent information in quite different ways, that allows you to ask some questions about the utility of different representations. These are important theoretical questions that you can now begin to address. I think highlighting this point is important. Currently, these two sections can read a bit like a laundry list of things you found out. I think binding them together with a bit more careful exposition would greatly improve these super important (and impressive) sections.

Some examples:

- Line 333 "To address questions about the dynamic stability of rings with higher embedding dimensions...".

- Why would I want to address this stability? Why is the embedding dimension of the ring an important question, and how is it relevant to biological neural systems?

- Line 287 "Next we explore the ability to embed rings in more than two dimensions"

- Again, why would you want to do this?

- Line 288 "At one limit..."

- Why are these limits the natural limits to consider? What are examples of neural systems that exhibit (or are hypothesized to exhibit) these limits?

A reader that has kept up with related literature would understand that this is a question that other researchers are attempting to address in empirical data, but many readers might not, and they would probably fail to appreciate what you are doing here.

These are excellent suggestions. We fully agree and have made several revisions to address these shortcomings.

In the section on high-D rings, the opening discussion about the ring length was not directly related to the properties of RNNs that implement such manifolds. Therefore, we moved this part and the corresponding plot (formerly Fig. 5d) to the Supplements (now Fig. S3d). Instead, we motivate this section by highlighting the relevance of higher dimensional manifolds in biological networks. Second, we realized that the procedure we used to construct the high-D rings was not explained carefully. We have remedied this shortcoming by completely rewriting that section. Third, and most importantly, we followed the reviewer’s advice and made use of our methodology to gain insights into the structure of the connectivity matrix associated with high-D rings. We found that the coupling that EMPJ imposes on networks is structurally related to the tuning curves of the units in the network. Specifically, we found that units with similar tuning are similarly coupled to other units in the network, and that the relationship between tuning and coupling weakens with the dimensionality of the ring manifold. These results have been added to Figure 5, and are shown below with the accompanying captions.

**Figure 5.** e) Left: The tuning similarity matrix measured in terms of the covariance of tuning functions for an RNN engineered to have O(2,6). Right: The coupling similarity matrix measured in terms of the inner product of the input weight vectors for all pairs of units for the same RNN. f) Violin plot showing the correlation coefficient of the tuning and coupling similarity matrices of engineered RNNs as a function of the ring’s embedding dimension, with no drift over the ring. The actual correlations are shown in black, and the values expected by chance, in red. To compute chance levels, we shuffled the label of tuning functions (i.e., the rows of the tuning similarity matrix) and recomputed the correlations.

We also fully rewrote the section on limitations of dynamical stability. We now provide a clear motivation (i.e., high-D rings have more curvature requiring faster changing gradients that may be more difficult to implement in an RNN). We have added new analyses and results to clarify the robustness of our results in panels b) and c) of Figure 6. Finally, we have revised the text to clarify how our work reveals important connections between the geometry and dynamics in RNNs.

Minor comments (suggestions, not required to fulfill):

Introduction

- I like describing this work as the inverse question of many existing approaches. To flesh that out, perhaps note how characterizing fixed points also relies on a linearization around fixed points, to understand existing structure. Here you are doing that to **build** the structure.

In an attempt to avoid jargon, we do not use the term “fixed points” in the Introduction. But to capture the main idea behind this comment, we now introduce our work as follows: “Here, we propose a novel analysis-by-synthesis approach that addresses the inverse problem of engineering RNNs that implement a desired latent variable model.” Moreover, early in the Results section, we highlight that, unlike previous work that used Jacobians to characterize dynamics, we use Jacobians to impose constraints on dynamics.

- Figure 1: Providing a little more detail about what "the task" is here would help to really guide a reader at this critical early stage. This task is pretty blank. Why not use the working memory task that you ultimately model? The task shown in the figure is never discussed again.

Since this figure was meant as a general introduction to the method, we didn’t want to limit it to the ring task. However, we agree that something more tangible would be helpful. Therefore, to address this comment, we have several revisions to the figure and caption. First, we made a schematic of the example task that is easy to understand (making a timed movement). We also labeled the axes in the second panel to reflect the actual latent variables of the task. The updated figure and captions are shown below.

**Figure 1. Embedding latent task dynamics into recurrent neural networks.** a) A hypothetical task, adapted from [[27]](https://paperpile.com/c/9BWY2G/zgsUH). The subject is instructed to produce a time interval by making a delayed movement. b) Latent task space over a manifold (gray rectangle) based on a clock-accumulator model [[28]](https://paperpile.com/c/9BWY2G/YJgm). The y-value reflects the speed of the clock, and the x-value reflects the state of the accumulator before movement initiation. In this model, the instruction sets the speed of the clock (length of the arrow) at which the accumulator evolves over time (faster for shorter intervals). Movement is initiated when the accumulator reaches a threshold (dashed line). c) A nonlinear embedding of the task manifold depicted in a neural state space spanned by the first three principal components of population activity. d) An RNN model to establish the desired nonlinear embedding.

Results

- The term "objective dynamics" is introduced in the first paragraph of the results without any real definition. It's somewhat clear to a reader more experienced with these ideas, and eventually becomes clear as one works through the text, but I think clarifying this up-front would be a better idea.

We have changed the phrase from “objective dynamics” to “target dynamics,” which we have clearly defined in the newly added introductory section of the Results.

- Line 106-107. Maybe just one more sentence here about why this is a sensible thing to do would be nice, especially for any reader not super familiar with dynamical systems theory.

We added a bit of motivation for why it makes sense to use the Jacobian: “In dynamical system analysis, Jacobians are typically used to assess local stability. Here, we exploit the relationship between Jacobians and local stability to impose constraints on network dynamics in the vicinity of the setpoint.”

- Equation 2: Why 1_N, not I for the identity?

Fixed.

- Line 120-121. Again, more hand holding would be nice, to explain why you can do this (essentially this is your point, you can replace J_RNN for J_OBJ, but now in the eigenbasis)

We have made several revisions in the text to make the ideas more accessible.

- Equation 6: define A and B here?

Fixed.

A ring attractor...

- The list of steps to perform the embedding seems more appropriate to explain during the general results section, since it's not special to the ring attractor example.

Unless the reviewer feels strongly about this, we prefer to keep the results focused and keep methodological details in Methods. We also note that although the list of steps is not special to the ring, our description includes numerous references to the ring as an example to help the reader understand the steps in the context of a specific objective.

- Line 168-171. Perhaps just re-define what is meant by radial and tangential eigenvectors here.

Fixed.

- Line 180-88, Re: non-zero mean paragraph is pretty unclear. I'm not exactly sure what the authors are saying here, and it definitely requires going to the supplemental material to understand what was done here at all. I would suggest expanding the explanation of what was done in the main text a bit, otherwise the reader will simply be lost.

We have rewritten this section entirely and made several clarifying adjustments. First, we replaced the phrase “non-zero mean” with “non-zero baseline,” which seems like a clearer choice of words. Second, we clarified our motivation for considering drift functions with non-zero baselines. Specifically, we began the section on the baseline with the following introduction: “While Jacobians can powerfully constrain the derivative of the drift function, they cannot be used to set a baseline drift because derivatives are insensitive to the baseline. [...] Creating a drift function with a non-zero baseline requires a non-trivial adjustment to the network synthesis procedure that goes beyond the use of Jacobians. Here, we demonstrate the solution in an example where we add different baselines to a sinusoidal drift function.

- Line 186 should be Figure 2c, not 1c.

Fixed.

Comparison...

- Line 231. That six fixed points minimize the error will certainly be noise dependent. I would recommend pointing this out, since you don't want to mistakenly imply to be making a more profound point than you are.

We have clarified that six fixed points minimizes error for the particular set of parameters that we tested.

Input control....

- Line 263. The authors refer to "the eigenvectors", but eigenvectors are referred to a few times in the last few sentences, and they are different eigenvectors each time. I would explain this a little more deliberately.

This section has been rewritten and is now clearer when referring to “the input subspace.”

Rings embedded...

- 305-307. "The eigendecomposition of the weight matrix..." I think I understand what the authors are saying here (because of the low rank form of the matrix, the dynamics go down to the low-D space and then back up) but perhaps some math here will really clarify this?

Sorry, our original text intended to explain what an eigendecomposition does, which is perhaps unnecessary. As part of our major revision in this section, we also addressed this point. The relevant section in the revised manuscript is modified as follows: “The rank of the connectivity matrix was the same as the number of embedding dimensions, independent of the drift function (Fig. 5d). This is a consequence of the fact that the data points we used to construct the system of linear equations had the same dimension as the number of population tuning functions.”

- If I understand what rings of "intermediate dimensionality"are, then 5a right is actually a bit misleading. In the case displayed, the dimensionality will be the same as the number of neurons (same as for 5a, left) because the number of latent tuning modes is the same. Obviously, this is the limit of latent modes, and will be equivalent to 5a left, except that each neuron is tuned to combinations of these modes, instead of having their own private "mode". To depict "intermediate dimensionality" there should be fewer modes (I believe). Perhaps this sub-panel needs a third figure showing the intermediate dimensionality, and then the limit of this (which is what is there now).

We agree that the figure is slightly misleading. We have adjusted the schematic to depict many neurons but only three modes. We have also made major revisions to make the figure less cluttered and more understandable.

- Line 314. "An infinitely broad von Mises function...". This took some serious mental visualization to understand the point. Why not just plot it in 5b?

We apologize for the lack of clarity. What we meant by infinitely broad was simply a flat tuning curve. However, in addressing this comment, we realized that there were additional details that we needed to explain. For example, the reason for the shift of the ring away from the equator of the hypersphere is that the flat tuning curve has a non-zero baseline (which is a consequence of our normalization scheme). On a more general level, the entire paragraph associated with Fig. 5d was solely based on a state-space analysis of high-dimensional ring geometries, and not its specific implementation by an RNN. Therefore, we decided to reorganize this section and move this panel to the Supplements as Fig. S3d). This revision also allowed us to streamline the main text, as requested by the reviewer’s earlier comments.

- Figure 5b, dimensions are c in the figure, but x in caption and main text.

Fixed.

Limitation of RNN

- Line 342. "Our method thus allows us to find the smallest network size...". Not really (although this is a specific technical quibble). It allows you to find out how poorly a network of a particular size performs, but you do not, for example, define a strict threshold for success, or a method for choosing the right number, other than just searching.

We fully agree and have changed the text accordingly.

- In figure 6b, what are the separate dots? Different networks?

Yes. We have clarified this in the caption.

––––––––––––––––––––––––––––––––––––––––––––––––––––––––––––––––––––––––––––

**Reviewer #2**

Main revisions:

- The success of the method relies on the hope that the nonlinear dynamics and the freedom in the weight matrix will succeed in reproducing the desired Jacobian through constraining it at a few select points that are high-dimensional expansions of points in the task manifold. The choice of these points could potentially improve or hurt the process, and yet it is not easy to give general guidance as to how to choose them. I think that the manuscript would be easier to read if this would be clarified early in the text, for example on lines 107-108, by stating that the Jacobian will only be constrained at a few points at which phi'(x) is evaluated. Besides a mild statement in the Method (lines 428-430), it looks like the issue of selecting the setpoints was not investigated much in the current draft. Is there an 'optimal' level of saturation of the units /scale of the projection vectors? Are there choices of the setpoints in the task manifold that make it harder to solve the dynamics --in particular, some setpoints seem to impose more structure in the weight matrix than others, like those situated on opposite branches of the star in Fig. 2b, which involves the same directions for the tangents U and the same eigenvalues, and yet different phi'(x). Are these specific points important to constrain, or does one gain extra freedom in the types of solutions for the weight matrix if one excludes these types of 'symmetric' points?

The reviewer brings up several good points about how the choice of setpoints may affect the solution of the eventual weight matrix.

1) As recommended by the reviewer, we clarify that the weight matrix is constrained by a choice of setpoints in the method description of the Results section, right before equation (6). To offer some intuition for how these points should be chosen, we have included a supplementary figure (S5) to include some exploration of how densely setpoints should be sampled from the manifold (shown below). Since we do not have a general theory for this question, we used an example of an 8-dimensional ring with 6 fixed points. We reference this figure in the Methods section, and it is shown with its caption below. For this example, we tested the lower bound of the number of setpoints, finding it to be around 12. Finally, we have also added a new section to the Methods to clarify the design considerations relevant to the “choice of setpoints.”

**Figure S5. Performance with respect to the number of setpoints.** We analyzed how the number of setpoints impacts the degree to which engineered RNNs can capture target manifolds and dynamics. We constructed the RNN to have O(8,6) with drift amplitude of 0.1 rad/s over a ring of radius 12. a) Violin plot showing the mean squared error between the measured and target drift function as a function of number of setpoints. Inset: Target drift function (red) and the corresponding measured drift function with 6 setpoints (black). b) Violin plot showing the deviation (equation 17) of neural states as a function of number of setpoints normalized by the radius. Results are shown for 20 simulations over a 5 second period, with deviation calculated from 20 evenly spaced initializations. Inset: The initial state (blue), intermediate states (gray), and end states (red) of a network constructed from 6 setpoints, plotted in a subspace spanned by the first three principal components.

2) Another point is raised about the level of saturation in the units. We found that network performance was improved when we increased the ring radius such that some of the units reached the saturation on the tanh() nonlinearity. However, if the radius is too large, performance also suffers. We have added a supplementary figure (S6) to illustrate this point (shown below). We have also added a new section to the Methods titled “Choice of embedding” to clarify the design considerations relevant to the saturation of the units.

**Figure S6. Ring radius, unit saturation, and deviation.** The radius of the ring is equal to the magnitude of the projection vectors. We quantified how the radius changes the operating point of units with respect to their saturation point, and impacts the robustness of a 400-unit RNN engineered for O(8,6). a) Projections of the first latent vector in the neural state space (abscissa) passed through a hyperbolic tangent function for rings with different radii (ordinate). In rings with large radii, more units operate near the saturation regime of their activation function. b) Violin plot showing the mean deviation (equation 17) as a function of radius. For each radius value, 30 networks were simulated. Mean deviation was computed across 20 trajectories over 2 seconds. The line shows the means of the distributions of deviations for each radius.

3) The point about symmetry in the setpoints is also interesting, and is now addressed in the Methods section. We used the tanh() function as the firing rate nonlinearity, and tanh(-x) = -tanh(x). Thus, if the manifold is symmetric about the origin, symmetry is imposed on the dynamics. Specifically, if symmetric points are on the manifold, then the drift function evaluated at each point must be equal and opposite. Consequently, two-dimensional rings with sinusoidal drift functions must have an even number of fixed points. For odd numbers of fixed points, there must be some kind of asymmetry introduced. Additionally, it becomes unnecessary to specify the dynamics at both points if their dynamics obey the imposed symmetry, since the linear constraints would be redundant. We have added a new section in the Methods titled “Choice of input-output nonlinearity” to clarify the design principles that must be considered for introducing asymmetries.

-Line 117, there is a mathematical expression: J_{RNN}=U \Sigma U^T , that is only valid if J_{RNN} is normal with real eigenvalues/eigenvectors, while the following equation (3) does not make any assumption about the properties of the eigenvectors or eigenvalues. I think it would be less confusing to write the more general equation J_{RNN}=U \Sigma U^(-1) on line 117. From what I understand, the J_{RNN} matrices that the authors constructed were not constrained to be normal (the statement made much later in the methods line 428 about the construction of the projection vectors from an orthogonal set does not necessarily imply that all eigenvectors of the final matrix are orthogonal, I think/verified). This point needs to be clarified. Also, the authors do not discuss how the choice of setting all eigenvalues to be real affects the solution -- purely decaying firing rates without oscillations can be somewhat unrealistic in the general case, and it would be desirable to discuss, or show, how the method could be extended to the case of complex eigenvalues.

Again, there are several important points to address here.

1) We fully agree with the point about using the more general form of the eigendecomposition and have revised the equation to reflect this. However, since we have used orthogonal eigenvectors in this paper, we clarified that assumption in the paragraph before equation (3) and kept all other equations the same.
2) The question about complex eigenvalues is very interesting and we are working on it. But we don’t have anything substantive to say about it yet. We have highlighted this point in the revised Discussion as an important extension for future research.

-Line 500-523: here, doesn't the construction method used -- which implicitly assumes that the dynamics can independently get to steady-state in the input dimension, while the ring's dynamics occur in the other dimensions -- strictly require orthogonality between the input direction and the directions along which the dynamics is not decaying very fast? It would be good to introduce/clarify this earlier, rather than just mentioning later (line 524) that the input direction is chosen to be orthogonal to the ring, which sounds as if this was not compulsory.

We have now clarified that the input control dimension is orthogonal to the ring in the main body of the text.

-Is there a reason why the authors chose to match the Jacobian to the task manifold, rather than more directly matching the projection of dx/dt on some vector matrix U (hence constraining the flow field as the authors somewhat do for fixed points in eq. 11)? It seems that, in this case, we can also use the expression: U(dx/dt)= (1/tau) U(-x+W^{T} phi(x)+I) to write an equation of the type: A_{x0} W = B_{x0} +C_{x0} where A, B and C are constrained by the desired properties of the matrix dx/dt at the particular point x0. I can see that for linear dynamical systems, the Jacobian has an advantage over dx/dt: it is independent of x; but it is not formally the case for the nonlinear systems considered by the authors. Is the optimization less sensitive to the choices of the setpoints if matching the Jacobian rather than dx/dt? It would be good to discuss this.

The reviewer is correct that it is possible to place constraints directly on dx/dt. Indeed, this is something we had already done when creating drift functions with shifted baselines by changing the location on the ring where dx/dt=0. In the revised draft, we have addressed the reviewer’s point in several ways. First, when introducing Jacobians, we offer more motivation for why it makes sense to use them for constraints while acknowledging that it is also possible to constrain the network dynamics directly. Second, when we introduce the baseline shifts, we make it more explicit that we are placing constraints on dx/dt. Finally, in the discussion we briefly discuss the pros and cons of using the Jacobian and offer examples where constraints on dx/dt might be more appropriate.

-I think it would help the readers if Fig. 5 would show examples of reconstructing the tuning curves from the RNN's activities initialized at different points on the task’s manifold. Also, in the associated text, it would be good to clearly expose and differentiate the two kinds of embedding that are implemented: first, an embedding of the ring in n-dimensions, and second the embedding of this n-dimensional representation in the N dimensional space of the units' activities.

We agree that visualizing a few examples of the tuning curves is useful. We decided to include them as a panel in Fig. S3, which is otherwise closely related to Fig. 5. This new panel is shown below. Regarding the second point about embedding, we have fully rewritten the relevant sections to clarify the steps we took to create high-D ring manifolds.

-It would be useful if the authors would display examples of weight matrices, and quickly investigate whether they seem similar to the weight matrices learned by backpropagation (e.g. similar to Cueva et al., ICLR 2019; by looking at whether units with similar preferred angle tend to wire positively together). This is a very easy step towards the goal, discussed by the authors, of comparing their networks to more classically trained RNNs.

Thank you for this suggestion. We have added new analyses along the lines suggested by the reviewer. Specifically, we asked whether RNN units with similar tuning functions are similarly driven by other units in the network. To answer this question, we analyzed the relationship between two similarity matrices, one characterizing the similarity between tuning functions of different units and the other characterizing the alignment between the input weight vectors driving different units. For the former, which we refer to as the *tuning similarity matrix*, we used the covariance between the tuning functions. In this matrix, element (i,j) contains the covariance between the tuning functions of the units i and j. For the latter, which we refer to as the *coupling similarity matrix*, we used the product of the connectivity matrix with its transpose. In this matrix, element (i,j) contains the inner product of the input weights into unit i and j. Finally, we asked whether these two matrices were similarly structured, i.e., whether the organization of tuning functions was similar to the organization of couplings.

We found that the covariance matrix of single-unit tuning curves is almost exactly the same as the weight matrix multiplied by its transpose for the 2-dimensional ring. The two similarity matrices were correlated for higher-dimensional rings as well, but the relationship was weakened with embedding dimension. This analysis is included in subplots of Fig. 5, shown below. We also cited the relevant work from Cueva et al. 2019.

**Figure 5.** e) Left: The tuning similarity matrix measured in terms of the covariance of tuning functions for an RNN engineered to have O(2,6). Right: The coupling similarity matrix measured in terms of the inner product of the input weight vectors for all pairs of units for the same RNN. f) Violin plot showing the correlation coefficient of the tuning and coupling similarity matrices of engineered RNNs as a function of the ring’s embedding dimension, with no drift over the ring. The actual correlations are shown in black, and the values expected by chance, in red. To compute chance levels, we shuffled the label of tuning functions (i.e., the rows of the tuning similarity matrix), and recomputed the correlations.

-Could the authors clarify if the decoding weights used to recover theta (which in the current manuscript are evaluated numerically) could be analytically expressed as a function of the projection vectors? It seems to me that, at least at fixed points, this should be feasible through (i) getting a relation between phi(x) and x by setting the derivative of x to 0; (ii) expressing x in the basis of the projection vectors; and (iii) using the known embedding relation between the projection vectors and [cos(theta), sin(theta)].

Yes, it should be possible to derive the decoding weights using the input weights. We note this as a possible alternative in the Methods section, right after equation (11). However, we also note that this approach may not work for rings with nonlinear embeddings in higher dimensions, which is why we used the decoding approach.

-Line 216: clarify 'infinitely' many, you mean as many as the number of units?

By “infinitely many” fixed points, we mean just a continuous ring attractor with no drift. We have clarified this in the text.

-Line 342: I guess you only mean that the process you just discussed gives the minimal size of the network capable of creating dynamics over a particular ring *when using your method to tune the weights*.

Our language here was slightly inaccurate. We have changed the text to reflect that our process allows us to quantify how well networks found through EMPJ perform. One could adjust the size to find the smallest network that performs above some threshold for success.

***************************

***Miscellaneous corrections***:

***************************

The paper needs to be reviewed for typos. Several examples are identified below.

Thank you for catching these; we have taken care of them.

-eq. 6: subindex should be 1 and not 0, to agree with line 140 'for some number m points'

Fixed, as are the references to point x_0 in Eq. 5 and line 140.

-fig 1 legend: 'MIddle left' -> 'Middle' (line 87) ; 'An RNN models' -> 'model' (line 91)

Fixed.

-line 117: should be a space after 'JRNN' before 'can'

Fixed.

-line 151: missing a 'to' in the sentence 'discrete fixed points can be used introduce error-correcting'

Fixed.

-fig 2a right: no input to the RNN, I think, in this figure.

Fixed.

-line 153: delete 'of' from 'When humans report of a previously…'

Fixed.

-line 182: missing 'similar' in 'adjustment to what' (-> ‘non-trivial adjustment similar to what we discussed previously’)

Fixed.

-line 185: grammar problem in the sentence 'a baseline can be added straightforwardly by an additional constraints to equation (6)…' for example, change 'an' to 'adding'

Fixed.

-line 186: typo in figure number 'Fig. 1c'? should be Fig. 2c

Fixed.

-line 263: add 'additional': 'the additional eigenvectors are the same as'

Fixed.

-line 279: 'red indicate' -> 'red colors indicate', or ‘red indicates’

Fixed.

-fig. 5b and lines 326-327: non-consistent mention of x and c as the coordinates of the sphere. Both of these symbols are already used to describe other variables in the text (the RNN dynamics for x and the tuning curves for c), so it would be desirable to use a different name for these coordinates (e.g. 'z')

To reduce any confusion, we have changed the figure caption and text to refer to the sphere dimensions as Dim 1, Dim 2, and Dim 3, and explained them in the caption.

-fig. 6a: what was the embedding dimension there?

The embedding dimension was 6. We have added this information into the caption.

-line 421: repeat that the 'setpoints' are the points where the jacobian will be specified

Fixed.

-line 440: add the word 'setpoints', for example, 'we first created a ring of setpoints by taking the cosine and sine of 64 evenly spaced values'

Fixed.

-line 450: extra = in the equation

Fixed.

-line 543: replace 'x' in the right-hand side by theta

Fixed.

-line 579: 'deviation from eq. 16', with 'deviation' typeset as math, reads better than 'deviation' typeset as text

Fixed.

-line 584: by 'number of the ring dimensionality', you mean 'the ring's spherical embedding dimensionality'? More generally, it would be good to make sure that throughout the text the spherical embedding vs. the embedding in the network's activities are labeled differently and clearly identified.

We have fixed this wording so that it reads “the ring’s embedding dimensionality.” We are unsure what the reviewer has in mind for the more general case. But, to minimize confusion regarding the embedding dimensionality, we introduced a notation of the form O(n_dim, n_fp) throughout the paper to refer to a ring embedded in n_dim dimensions with n_fp fixed points.

-The typesetting of the equations is not always consistent, cf. ’Jobj’ in line 109 and 120, or the 'deviation' measure which is at times written as text. Ultimately, fixing this can make the text nicer to read.

We have made the equation typesetting more consistent. The mentioned examples are both fixed, and we have looked for other examples where there are inconsistencies. It is our intention to make further adjustments to the math typesetting after the final review and in accordance with the journal’s formatting requirements.

In addition to addressing the specific concerns of the reviewers listed above, we also made changes to improve the overall clarity and appearance of the paper. Below, we have listed some changes to the figures, methods, and supplementary figures that are notable.

1. Figures have been revised to have more consistent formatting, clearer labeling of axes and subplots, and more informative captions.
2. In all cases where we had used performance metrics for engineered networks (e.g., deviation as a function of embedding dimension), we have now included the corresponding floor and/or ceiling value of the relevant metric based on shuffled data.
3. In Methods, we have added a few subsections focused on design considerations that can help EMPJ function more smoothly. These subsections include “Choice of setpoints,” “Choice of input-output nonlinearity,” “Choice of embedding,” and ”Solving the linear equations.”
4. In Methods, we have added a subsection on “Simulations in the presence of noise (diffusion)” describing the details of how noise was added to RNN simulations. We have also added a “Bias/variance comparison” subsection where we explain how we quantify performance statistics of the engineered networks.
5. In Methods, we have revised and simplified the equations so that they can be straightforwardly replicated.
6. In the Supplementary Figures, we have added results (Fig. S1) on the success of our method under different conditions, which we had previously alluded to but had not shown. The figure is shown below with its caption.
7. To simplify the text and clearly state the parameters used in every simulation, we have introduced a notation of the form O(n_dim, n_fp) throughout the paper to refer to a ring embedded in n_dim dimensions with n_fp fixed points.

**Reviewers’ full comments**

–––––––––––––––––––––––––––––––––––––––––––––––––––––––––––––––––––––––––––

**Reviewer #1**

Summary:

The authors present a generally clearly-written study of a novel method for constructing artificial neural networks. The method solves for the recurrent connectivity by equating the linearized network dynamics about a set of network states to the linearized dynamics of a desired task (e.g. ring attractor). By choosing many such points, the connectivity can be sufficiently constrained. The authors use the method to construct a network model of a ring attractor with a set of discrete fixed points, and illustrate an equivalence of the network model to drift diffusion models (e.g. studying bias-variance tradeoff in these models). The authors go on to embed rings of more complex geometry into their networks, and examine the properties of these networks as they relate to information representation in neural systems.

Minor revisions:

1. One question that many readers will want to know the answer to is how robust the method is, a point which the authors note twice themselves (line 212 "such large vectors caused unpredictable network behavior" and line 397 "if the dynamics are fast enough that linear approximations no longer work"). Obviously, answering this question will be dependent on the task, number of neurons, etc. so this can't be answered exhaustively, but I think just giving the reader a taste of the robustness is important. For example, answering that question within the confines of the ring attractor model, with whatever standard parameters were used throughout the paper would be sufficient. Two ways I could imagine answering this question would be to measure the relaxation dynamics to increasingly larger levels of internal noise (as was already done), or to measure the relaxation dynamics to initializations way off the ring (although I leave it to the authors to decide the best way to illustrate this point).

One additional benchmark that might be useful would be to compare the robustness to noise or initialization off-manifold to methods (such as NEF) that use the global dynamics equations (as the authors noted). This might be a more expansive revision that the authors are willing to undertake. I leave it to the authors to decide if they wish to include this, but I personally feel it would strengthen the paper and better allow readers to understand the relationship of this new method to existing methods.

General comments:

Broadly, the paper is well-written and clear. However, also, broadly, I would categorize the writing as being targeted to readers with a high-level of prior knowledge about the subject. Personally, I would urge the authors to "hand-hold" the reader a bit more, to increase the chances that a reader without a super high-level of subject familiarity will read and understand this very important work. (This if merely a suggestion, not a requirement). It's difficult to give an exhaustive lists of these issues, but I will provide two, to hopefully clarify this:

1). The introduction can be a bit jargony (e.g. notice the number of quoted phrases- "latent task space", "latent task manifold", "latent task dynamics"). This is not a problem for a fairly acquainted reader, but the authors might want to unpack some of these ideas a bit more.

2). The last two sections are pretty unmotivated and I was often left wanting more exposition here ("Rings embedded..." and "Limitations of RNN..."). To me, this is unfortunate because here you are really paying off the development of your method- you can use it to construct neural systems that represent information in quite different ways, that allows you to ask some questions about the utility of different representations. These are important theoretical questions that you can now begin to address. I think highlighting this point is important. Currently, these two sections can read a bit like a laundry list of things you found out. I think binding them together with a bit more careful exposition would greatly improve these super important (and impressive) sections.

Some examples:

- Line 333 "To address questions about the dynamic stability of rings with higher embedding dimensions...".

- Why would I want to address this stability? Why is the embedding dimension of the ring an important question, and how is it relevant to biological neural systems?

- Line 287 "Next we explore the ability to embed rings in more than two dimensions"

- Again, why would you want to do this?

- Line 288 "At one limit..."

- Why are these limits the natural limits to consider? What are examples of neural systems that exhibit (or are hypothesized to exhibit) these limits?

A reader that has kept up with related literature would understand that this is a question that other researchers are attempting to address in empirical data, but many readers might not, and they would probably fail to appreciate what you are doing here.

Minor comments (suggestions, not required to fulfill):

Introduction

- I like describing this work as the inverse question of many existing approaches. To flesh that out, perhaps note how characterizing fixed points also relies on a linearization around fixed points, to understand existing structure. Here you are doing that to **build** the structure.

- Figure 1: Providing a little more detail about what "the task" is here would help to really guide a reader at this critical early stage. This task is pretty blank. Why not use the working memory task that you ultimately model? The task shown in the figure is never discussed again.

Results

- The term "objective dynamics" is introduced in the first paragraph of the results without any real definition. It's somewhat clear to a reader more experienced with these ideas, and eventually becomes clear as one works through the text, but I think clarifying this up-front would be a better idea.

- Line 106-107. Maybe just one more sentence here about why this is a sensible thing to do would be nice, especially for any reader not super familiar with dynamical systems theory.

- Equation 2: Why 1_N, not I for the identity?

- Line 120-121. Again, more hand holding would be nice, to explain why you can do this (essentially this is your point, you can replace J_RNN for J_OBJ, but now in the eigenbasis)

- Equation 6: define A and B here?

A ring attractor...

- The list of steps to perform the embedding seems more appropriate to explain during the general results section, since it's not special to the ring attractor example.

- Line 168-171. Perhaps just re-define what is meant by radial and tangential eigenvectors here.

- Line 180-88, Re: non-zero mean paragraph is pretty unclear. I'm not exactly sure what the authors are saying here, and it definitely requires going to the supplemental material to understand what was done here at all. I would suggest expanding the explanation of what was done in the main text a bit, otherwise the reader will simply be lost.

- Line 186 should be Figure 2c, not 1c.

Comparison...

- Line 231. That six fixed points minimize the error will certainly be noise dependent. I would recommend pointing this out, since you don't want to mistakenly imply to be making a more profound point than you are.

Input control....

- Line 263. The authors refer to "the eigenvectors", but eigenvectors are referred to a few times in the last few sentences, and they are different eigenvectors each time. I would explain this a little more deliberately.

Rings embedded...

- 305-307. "The eigendecomposition of the weight matrix..." I think I understand what the authors are saying here (because of the low rank form of the matrix, the dynamics go down to the low-D space and then back up) but perhaps some math here will really clarify this?

- If I understand what rings of "intermediate dimensionality"are, then 5a right is actually a bit misleading. In the case displayed, the dimensionality will be the same as the number of neurons (same as for 5a, left) because the number of latent tuning modes is the same. Obviously, this is the limit of latent modes, and will be equivalent to 5a left, except that each neuron is tuned to combinations of these modes, instead of having their own private "mode". To depict "intermediate dimensionality" there should be fewer modes (I believe). Perhaps this sub-panel needs a third figure showing the intermediate dimensionality, and then the limit of this (which is what is there now).

- Line 314. "An infinitely broad von Mises function...". This took some serious mental visualization to understand the point. Why not just plot it in 5b?

- Figure 5b, dimensions are c in the figure, but x in caption and main text.

Limitation of RNN

- Line 342. "Our method thus allows us to find the smallest networks size...". Not really (although this is a specific technical quibble). It allows you to find out how poorly a network of a particular size performs, but you do not, for example, define a strict threshold for success, or a method for choosing the right number, other than just searching.

- In figure 6b, what are the separate dots? Different networks?

–––––––––––––––––––––––––––––––––––––––––––––––––––––––––––––––––––––––––

**Reviewer #2**

This is a very interesting manuscript that describes a new method for generating interpretable neural networks when the flow field of the dynamics can be specified. While the paper is well-motivated and shows convincing numerical results, I think that it requires a few clarifications.

*******************

***Main comments***:

*******************

-The success of the method relies on the hope that the nonlinear dynamics and the freedom in the weight matrix will succeed in reproducing the desired Jacobian through constraining it at a few select points that are high-dimensional expansions of points in the task manifold. The choice of these points could potentially improve or hurt the process, and yet it is not easy to give general guidance as to how to choose them. I think that the manuscript would be easier to read if this would be clarified early in the text, for example on lines 107-108, by stating that the Jacobian will only be constrained at a few points at which phi'(x) is evaluated. Besides a mild statement in the Method (lines 428-430), it looks like the issue of selecting the setpoints was not investigated much in the current draft. Is there an 'optimal' level of saturation of the units /scale of the projection vectors? Are there choices of the setpoints in the task manifold that make it harder to solve the dynamics --in particular, some setpoints seem to impose more structure in the weight matrix than others, like those situated on opposite branches of the star in Fig. 2b, which involve the same directions for the tangents U and the same eigenvalues, and yet different phi'(x). Are these specific points important to constrain, or does one gain extra freedom in the types of solutions for the weight matrix if one excludes these types of 'symmetric' points?

-Line 117, there is a mathematical expression: J_{RNN}=U \Sigma U^T , that is only valid if J_{RNN} is normal with real eigenvalues/eigenvectors, while the following equation (3) does not make any assumption about the properties of the eigenvectors or eigenvalues. I think it would be less confusing to write the more general equation J_{RNN}=U \Sigma U^(-1) on line 117. From what I understand, the J_{RNN} matrices that the authors constructed were not constrained to be normal (the statement made much later in the methods line 428 about the construction of the projection vectors from an orthogonal set does not necessarily imply that all eigenvectors of the final matrix are orthogonal, I think/verified). This point needs to be clarified. Also, the authors do not discuss how the choice of setting all eigenvalues to be real affects the solution -- purely decaying firing rates without oscillations can be somewhat unrealistic in the general case, and it would be desirable to discuss, or show, how the method could be extended to the case of complex eigenvalues.

-Line 500-523: here, doesn't the construction method used -- which implicitly assumes that the dynamics can independently get to steady-state in the input dimension, while the ring's dynamics occur in the other dimensions -- strictly require orthogonality between the input direction and the directions along which the dynamics is not decaying very fast? It would be good to introduce/clarify this earlier, rather than just mentioning later (line 524) that the input direction is chosen to be orthogonal to the ring, which sounds as if this was not compulsory.

-Is there a reason why the authors chose to match the Jacobian to the task manifold, rather than more directly matching the projection of dx/dt on some vector matrix U (hence constraining the flow field as the authors somewhat do for fixed points in eq. 11)? It seems that, in this case, we can also use the expression: U(dx/dt)= (1/tau) U(-x+W^{T} phi(x)+I) to write an equation of the type: A_{x0} W = B_{x0} +C_{x0} where A, B and C are constrained by the desired properties of the matrix dx/dt at the particular point x0. I can see that for linear dynamical systems, the Jacobian has an advantage over dx/dt: it is independent of x; but it is not formally the case for the nonlinear systems considered by the authors. Is the optimization less sensitive to the choices of the setpoints if matching the Jacobian rather than dx/dt? It would be good to discuss this.

-I think it would help the readers if Fig. 5 would show examples of reconstructing the tuning curves from the RNN's activities initialized at different points on the task’s manifold. Also, in the associated text, it would be good to clearly expose and differentiate the two kinds of embedding that are implemented: first, an embedding of the ring in n-dimensions, and second the embedding of this n-dimensional representation in the N dimensional space of the units' activities.

-It would be useful if the authors would display examples of weight matrices, and quickly investigate whether they seem similar to the weight matrices learned by backpropagation (e.g. similar to Cueva et al., ICLR 2019; by looking at whether units with similar preferred angle tend to wire positively together). This is a very easy step towards the goal, discussed by the authors, of comparing their networks to more classically trained RNNs.

-Could the authors clarify if the decoding weights used to recover theta (which in the current manuscript are evaluated numerically) could be analytically expressed as a function of the projection vectors? It seems to me that, at least at fixed points, this should be feasible through (i) getting a relation between phi(x) and x by setting the derivative of x to 0; (ii) expressing x in the basis of the projection vectors; and (iii) using the known embedding relation between the projection vectors and [cos(theta), sin(theta)].

-Line 216: clarify 'infinitely' many, you mean as many as the number of units?

-Line 342: I guess you only mean that the process you just discussed gives the minimal size of the network capable of creating dynamics over a particular ring *when using your method to tune the weights*.

***************************

***Miscellaneous corrections***:

***************************

The paper needs to be reviewed for typos. Several examples are identified below.

-eq. 6: subindex should be 1 and not 0, to agree with line 140 'for some number m points'

-fig 1 legend: 'MIddle left' -> 'Middle' (line 87) ; 'An RNN models' -> 'model' (line 91)

-line 117: should be a space after 'JRNN' before 'can'

-line 151: missing a 'to' in the sentence 'discrete fixed points can be used introduce error-correcting'

-fig 2a right: no input to the RNN, I think, in this figure.

-line 153: delete 'of' from 'When humans report of a previously…'

-line 182: missing 'similar' in 'adjustment to what' (-> ‘non-trivial adjustment similar to what we discussed previously’)

-line 185: grammar problem in the sentence 'a baseline can be added straightforwardly by an additional constraints to equation (6)…' for example, change 'an' to 'adding'

-line 186: typo in figure number 'Fig. 1c'? should be Fig. 2c

-line 263: add 'additional': 'the additional eigenvectors are the same as'

-line 279: 'red indicate' -> 'red colors indicate', or ‘red indicates’

-fig. 5b and lines 326-327: non-consistent mention of x and c as the coordinates of the sphere. Both of these symbols are already used to describe other variables in the text (the RNN dynamics for x and the tuning curves for c), so it would be desirable to use a different name for these coordinates (e.g. 'z')

-fig. 6a: what was the embedding dimension there?

-line 421: repeat that the 'setpoints' are the points where the jacobian will be specified

-line 440: add the word 'setpoints', for example, 'we first created a ring of setpoints by taking the cosine and sine of 64 evenly spaced values'

-line 450: extra = in the equation

-line 543: replace 'x' in the right-hand side by theta

-line 579: 'deviation from eq. 16', with 'deviation' typeset as math, reads better than 'deviation' typeset as text

-line 584: by 'number of the ring dimensionality', you mean 'the ring's spherical embedding dimensionality'? More generally, it would be good to make sure that throughout the text the spherical embedding vs. the embedding in the network's activities are labeled differently and clearly identified.

-The typesetting of the equations is not always consistent, cf. ’Jobj’ in line 109 and 120, or the 'deviation' measure which is at times written as text. Ultimately, fixing this can make the text nicer to read.
