# Supplementary material for: Engineering recurrent neural networks from task-relevant manifolds and dynamics
Source: PLoS Comput Biol. 2020 Aug 12;16(8):e1008128. doi: 10.1371/journal.pcbi.1008128 (PMC7446915; doi:10.1371/journal.pcbi.1008128)
Supplement: Attachment — Submitted filename: EP_MJ_ploscompbio_secondresponse.docx [file pcbi.1008128.s009.docx]

We thank the reviewers for their comments on our revised draft. We have made a few additional changes in response to comments made by Reviewer #2, which are confined to the section titled “Rings embedded in high-dimensional space” and highlighted in the submitted draft. Below, we give a detailed response to the reviewer comments, which are shown in blue.

Reviewer #1:

All of my initial concerns have been addressed by the authors. In particular, I think that the rewrite of several later sections ("Rings embedded..." and "Limitations of RNN...") has greater improved the clarity of the work.

We thank the reviewer for this comment, and are glad they feel that the text’s clarity has been improved.

---------------------------------------------------------------------------------------------------

Reviewer #2:

The authors have made a considerable amount of additional analyses and text clarifications which, in my opinion, now leads to an even greater article that is really nice to read.

We thank the reviewer for this kind comment.

My only minor remaining thought is that I was left partially unsatisfied with the analysis in Fig. 5e-f, though I want to leave it to the authors to decide whether they agree with my concerns and therefore want to improve this analysis in the final manuscript. Indeed, it is relatively expected that units with similar input weights would have similar tuning curves, as the input shapes the tuning. More interesting to me would be whether units with similar *output* weight vectors have similar tuning curves -- this would be expected in a 2d ring as the connectivity is likely symmetric, but for larger embedding dimensions it is unclear what happens. To answer this question, the analysis in Fig. 5e-f could be repeated using an 'output coupling' similarity matrix (J^T J) rather than the input coupling similarity matrix (J J^T) used by the authors. Alternatively, some examples of weight matrices could be presented with units ordered as for the sorted tuning similarity matrix.

We agree that the analysis of Fig. 5e-f could have been more thorough. As the reviewer mentioned, it is not very surprising that the tuning similarity is similar to what we referred to as “coupling similarity.” We therefore extended our analysis with the “ouptut coupling matrix” suggested by the reviewer, and changed the term “coupling similarity matrix” to “input coupling matrix” to be more consistent. Since we now had three matrices to compare, we also introduced a partial correlation analysis, controlling for the third variable for each correlation pair.

To answer the reviewer’s question, we did find that there was a correlation between tuning similarity and output coupling. However, this almost disappeared when controlling for input coupling, suggesting that symmetry in the weight matrix is primarily responsible for any correlation between output coupling and tuning similarity. We interpreted this result using a causal graph, shown in panel g). In the text accompanying the figure, we use this framework to explain each of the correlation results.

In addition, the method used to sort the matrices in 5e should be indicated (for the analysis, it would make sense that some statistical technique such as hierarchical clustering would be used to sort one of them, and the second one would then also follow this order suggested by the data of the first one).

We have clarified in the caption for Fig. 5e that all three matrices are ordered by the preferred orientation of the tuning curves.

For reference, below we show the previous and current versions of the panels and captions that were changed.

**Figure 5. Embedding rings in higher dimensions.** e) Left: The tuning similarity matrix measured in terms of the covariance of tuning functions for an RNN engineered to have O(2,6). Right: The coupling similarity matrix measured in terms of the inner product of the input weight vectors for all pairs of units for the same RNN. f) Violin plot showing the correlation coefficient of the tuning and coupling similarity matrices of engineered RNNs as a function of the ring’s embedding dimension, with no drift over the ring. The actual correlations are shown in black, and the values expected by chance, in red. To compute chance levels, we shuffled the label of tuning functions (i.e., the rows of the tuning similarity matrix), and recomputed the correlations.

Updated version of Fig. 5 e-g):

**Figure 5. Embedding rings in higher dimensions.** e) Left: The tuning similarity matrix measured as the covariance of tuning functions for an RNN engineered to have O(2,6). Middle: The input coupling matrix measured as the inner product of the input weight vectors for all pairs of units for the same RNN. Right: The output coupling matrix measured as the inner product of the output weight vectors for all pairs of units for the same RNN. Matrix indices are ordered by the preferred orientation of the tuning curves. f) Left: Violin plot of the correlation coefficient between the elements of the tuning similarity and input coupling matrices of engineered RNNs. The results are shown for various ring embedding dimensions with no drift over the ring. The actual correlations are shown in black, partial correlations controlling for the output coupling matrix in blue, and the values expected by chance in red. To compute chance levels, we shuffled the label of tuning functions (i.e., the rows of the tuning similarity matrix) and recomputed the correlations. Middle: Same analysis repeated for the tuning similarity and output coupling matrices, with the partial correlations controlling for the input coupling matrix. Right: Same analysis repeated for the input coupling and output coupling matrices, with the partial correlations controlling for the tuning similarity matrix. g) Causal graph showing the relationship between the input coupling matrix (left node),  output coupling matrix (right node), tuning similarity matrix (bottom node), and the weight matrix (top node). The three dashed lines marked as i, ii, and iii correspond to the three correlation plots in panel f (left to right).
